# The cancer–microbiome axis: Mechanisms and emerging therapeutic strategies

**DOI:** 10.1007/s00281-026-01072-1

**Published:** 2026-04-16

**Authors:** Maria Rae Walker, Marlene Schwarzfischer, Michael Scharl

**Affiliations:** Department of Gastroenterology and Hepatology, University Hospital Zurich, University of Zurich, Rämistrasse 100, 8091 Zurich, Switzerland

**Keywords:** Cancer microbiome, Tumor immunity, Fecal microbiota transplantation (FMT), Bacterial immunomodulation, Immune checkpoint inhibitors, Microbiome-based therapeutics

## Abstract

The human microbiome has emerged as a critical modulator of cancer development, progression, and therapeutic response. Advances in sequencing and functional profiling have revealed that commensal microorganisms—particularly bacteria—interact closely with host immune and metabolic pathways, influencing tumor immunity across multiple cancer types. Dysbiosis of the gut microbiome has been associated with tumorigenesis, immune evasion, and resistance to therapy, while specific microbial taxa and metabolites have been shown to enhance antitumor immune responses. These discoveries have catalyzed the development of microbiome-based therapeutic strategies aimed at reshaping host–tumor interactions. This review summarizes current understanding of the cancer–microbiome axis, with a particular focus on therapeutic interventions that leverage microbial modulation. We discuss fecal microbiota transplantation (FMT) as an early, proof-of-concept approach demonstrating the capacity of the microbiome to restore responsiveness to immune checkpoint inhibitors, while also highlighting its limitations related to variability, standardization, and mechanistic uncertainty. We then examine emerging reductionist strategies, including supplementation with individual bacterial strains, defined consortia, and engineered microbes designed to deliver immunomodulatory payloads directly within the tumor microenvironment. Finally, we explore how microbial modulation interfaces with conventional cancer therapies such as chemotherapy, hormonal therapy, and cellular immunotherapies. Together, these studies illustrate a rapidly evolving field transitioning from correlative observations to mechanistically informed therapeutic design. While significant technical and biological challenges remain, continued integration of microbiology, immunology, and synthetic biology holds promise for translating microbiome-based interventions into safe, precise, and effective cancer therapies.

## Introduction

One of the first disciplines of modern biology to emerge was microbiology, which underwent a period of methodological and conceptual advancement during the mid-19th century. Early work by Louis Pasteur and Robert Koch focused predominantly on individual bacteria and pathogenic organisms; however, the study of microorganisms coexisting within the human body remained largely unexplored for over a century. Only recently was it recognized that diseases, previously thought to be unrelated to microbial influence may, in fact, be linked to alterations to the healthy human microbiome [1]. Gastrointestinal diseases, such as inflammatory bowel disease, but also metabolic disorders like type-2 diabetes have been shown to be affected by changes in our gut microbiome, illustrating the distal influences of these host microbes [2]. The past few decades have opened a new era in disease research: highlighting how an enormous ecosystem of microbial genes and metabolites plays intricate roles in the progression of non-infectious diseases. Of particular note is the emerging evidence implicating the microbiome in cancer development, diagnosis, and even progression. As we continue to unravel these complex host-microbe interactions, a new therapeutic frontier is emerging in which microbes themselves may be harnessed as agents of cancer treatment. This review summarizes key studies pursuing this concept, and discusses future avenues for advancement in this rapidly evolving field.

## Current state of the art

### Dysbiosis

**Fig. 1:**
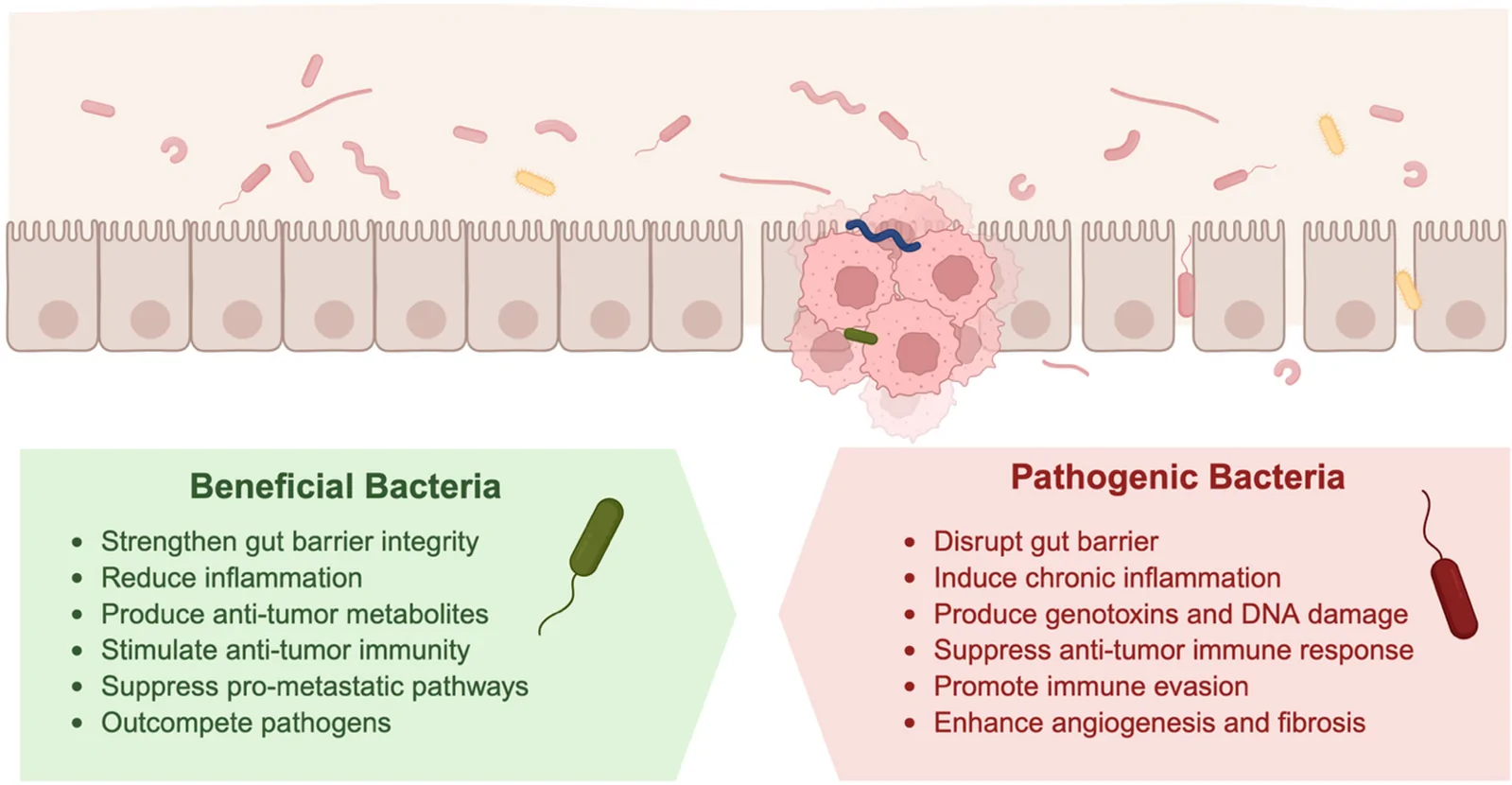
The oncobiome and cancer progression: pathogenic bacteria promote tumor progression, while beneficial bacteria support anti-tumor immunity

The human microbiome has recently been recognized as an emerging enabling characteristic in the updated Hallmarks of Cancer as a contributing mechanism of cancer progression [3]. The intestinal microbiome, which houses the largest number of microorganisms in the human body, is often used as a biomarker for cancer pathogenesis, as its composition tends to shift toward dysbiosis during disease [4]. Remarkably, the human body is composed of nearly equal numbers of microbial and human cells, underscoring the profound integration of microbial life within our physiology [5]. Encompassing the entire community of microorganisms, the human microbiome resides predominantly on mucosal surfaces and functions symbiotically with our host cells. Although the microbiome includes bacterial, viral, fungal, and protozoan species, this review focuses primarily on the bacterial compartment, as it represents the majority of current microbiome research. Perturbations of healthy microbial populations can create conditions and foster an environment that promotes tumorigenesis [6]. This occurs, by one mechanism, through disruption of the epithelial barrier integrity, which allows for microbes or microbial products to translocate across the gut and generate localized and systemic inflammation. Other mechanisms such as the cross-talk of microbial species with the host immunity, metabolic secretions altering homeostatic substrate levels, and the presence of genotoxins arising from microbial imbalances also drive the initiation of cancer from the microbiome site [7]. The identification therefore of more beneficial versus pathogenic bacteria in the context of cancer progression is an important step to understanding and preventing these mechanisms from occurring (Fig. 1.). Stool samples from patients with gastrointestinal cancers often display reduced alpha-diversity (i.e. overall bacterial species richness) and a relative enrichment of undesirable and potentially pathogenic taxa. For instance, colorectal cancer progression is often associated with elevated abundance of the genus *Fusobacterium*, which can outcompete and diminish other beneficial bacterial populations. Microbial dysbiosis is observed in numerous other diseases, and its specific contribution in cancer development and progression continue to be actively investigated.

### Methods of detection

Modern microbiology benefits from exceptionally more sophisticated tools for bacterial identification and analysis. Studies investigating changes in bacterial composition commonly employ next-generation sequencing (NGS) metagenomic techniques [8]. Amplicon sequencing (most often 16 S rRNA sequencing) uses a complementary DNA library to identify known bacterial taxa present within a sample. This polymerase chain reaction (PCR)-based method is cost-effective and robust but provides limited resolution at the strain level and provides little functional insight. In contrast, shotgun metagenomics sequences all DNA present in a sample, enabling higher taxonomic resolution and information about functional pathways. These two methods remain the most widely used, and the choice between them depends on research objectives and budget constraints. Less commonly used, metatranscriptomics analyzes microbial RNA transcripts to capture active gene expression, offering a more accurate representation of microbial activity. More recently, and more extensively, spatial transcriptomics approaches have begun to incorporate bacterial signatures, providing valuable spatial context regarding microbial localization within tissues.

Beyond sequencing, in vitro and in vivo detection methods remain essential techniques for advancing our understanding of microbial presence and behavior within host tissues. Notably, few non-NGS methods are considered both consistent and robust for detecting bacteria within eukaryotic tissues, making this a critical area for methodological improvement. Two-dimensional histological staining can visualize bacteria in tissue sections using fluorescently tagged antibodies or fluorescence in situ hybridization (FISH) targeting bacterial components such as lipopolysaccharides (LPS) or 16 S RNA. However, routine techniques capable of distinguishing viable bacteria from dead or fragmented ones are still lacking. Culture-based methods can achieve this distinction but are usually low-throughput and depend on prior knowledge of specific cultivation conditions. A more recent, and much more high-throughput approach, termed culturomics combines optimized culture conditions with MALDI-TOF mass spectrometry to identify microorganisms that can even be previously unknown. With each bacterial species having a unique protein fingerprint, this technique is accurate and reproducible while allowing broad coverage of culturable microorganisms [9].

New approaches are being developed to address these challenges, including methods that selectively detect only metabolically active or proliferating bacteria. For instance, labeled enzymatic substrates of bacterial metabolites can be used to visualise living bacterial populations [10]. More recently, phage-based technologies have utilized targeted bacteriophages to infiltrate exclusively living bacteria to modify or label them [11]. Another innovative approach, INVADEseq, integrates single-cell RNA sequencing methods with specialized primers targeting bacterial 16 S rRNA, thereby generating libraries that capture both eukaryotic and bacterial transcripts simultaneously [12].

Overall, while current microbiome research provides powerful tools for profiling bacterial communities, improved techniques are needed to study how live bacteria interact with and migrate within their eukaryotic hosts. Furthermore, analysis pipelines for large metagenomic datasets often remain inconsistent across studies. Recognizing and addressing this variability is critical for advancing the field in a standardized and scientifically rigorous manner [13].

## The cancer-microbiome axis

### Tumor microbiome

With the growing recognition of the microbiome’s involvement in tumor progression, research has begun to focus on the existence and function of a localized tumor microbiome. This refers to specific bacterial niches that are compositionally and functionally distinct from adjacent healthy tissue. Unlike systemic alterations arising from dysbiosis in distal organs, tumor microbiomes represent a presence of unique bacterial communities within the tumor microenvironment [14].

A pivotal study published in 2020 analyzed more than 1,500 tumors, comparing bacterial content in tumor tissues and adjacent healthy controls, revealing distinct microbial compositions between these compartments [15]. Most detected bacterial species were intracellular, interacting with both tumor and immune cells within this unique niche. Specific characteristics of the tumor microenvironment appear to facilitate microbial localization. Solid tumors often contain regions of low oxygen due to their hypoxic nature, that favour the growth of anaerobic bacteria. Moreover, the high metabolic activity of tumor cells provides a rich nutrient source, while the inherent immunosuppressive environment allows for bacteria to evade host immune surveillance and elimination. With modern detection technologies capable of identifying low-abundance DNA, these findings have opened a new avenue of investigation into the specific presence and function of cancer-promoting bacteria in close association with the tumor tissue [16]. Furthermore, the potential translocation of microbes from their typical habitats to these atypical tumor sites warrants further investigation to elucidate the underlying mechanisms driving this phenomenon [17].

### Immune interactions

The healthy human microbiome maintains constant communication with its host to sustain physiological homeostasis. The recognition and discrimination between healthy commensal species and pathogenic ones is crucial for a functioning sampling of our outside world, while gaining immune tolerance to our native species. Despite major advances, significant gaps remain in our understanding of host-microbiome interactions in both health and disease. For instance, the exact mechanisms in which the immune system establishes the eloquent balance of tolerance to our host microbiome while also able to identify pathogenic species is still being explored; and the specifics, such as with which molecular receptors are these outcomes achieved, are still largely unknown [18]. Continued investigation of these interfaces is essential to elucidate their roles in tumor biology.

Due to their systemic circulation, immune cells serve as effective carriers of microbial signals to distant sites. Pattern-recognition receptors (PRRs) on immune cells detect bacterial components, primarily at mucosal barriers, and transmit these signals to other immune subsets. Although these interactions occur at multiple mucosal surfaces, the gut and corresponding gut-associated lymphoid tissues (GALTs) remain the largest area of cross-talk between the microbiome and the inside body. Additionally, secreted bacterial molecules can directly modulate immune responses [19, 20]. Numerous metabolites secreted by commensal microbes have been shown to modulate immune activity, influencing cancer development and the tumor microbiome. These mechanisms will be discussed in detail in later sections [21, 22]. Collectively, these studies highlight the capacity of microbiome-derived molecules to directly regulate immune system function.

### Prognosis

The identification of specific bacterial species as biomarkers for cancer prognosis represents a logical initial step in developing targeted microbiome-based therapies. With the widespread accessibility of NGS tools and the global availability of large public datasets, it has become increasingly feasible to identify microbial taxa associated with particular cancer outcomes [23, 24]. By correlating these bacteria with clinical parameters such as overall survival or treatment response, researchers can prioritize functional studies aimed at exploring causal mechanisms.

Interestingly, only one bacterial species is formally recognized as a carcinogen by the International Agency for Research on Cancer (IARC): *Helicobacter pylori.* This organism is linked to upper gastrointestinal malignancies, including gastric adenocarcinoma and MALT lymphomas, with an incidence rate of 8.7 cases per 100,000 individuals per year [25, 26]. Other bacteria taxa have been correlated with poor cancer prognosis but have not yet been officially classified as pro-tumorigenic. However, as large patient cohorts continue to be analyzed, additional species are being identified in association with cancer incidence and progression [27, 28]. At present, the correlation between most identified bacterial species and their functional effects on cancer remain largely descriptive and lack consistent casual evidence. Nevertheless, the collective findings from these studies contribute valuable insights that are gradually assembling a more comprehensive picture of how the microbiome influences cancer pathogenesis.

## Microbial therapies for cancer

As our understanding of microbe-disease associations expands, and given that certain bacterial species are consistently detected at sites of pathogenesis, it is a logical progression to explore the potential of bacteria – or their secreted products – as therapeutic agents in cancer [29]. The mechanisms underlying these microbial influences can broadly be categorized into two types: those involving direct interactions between bacteria and with the cancer associated tissues (including immune cells), and those mediated by the secretion of microbial proteins or metabolites that drive downstream pathogenesis. A thorough understanding of these mechanisms is essential for the rational design of microbial-based therapeutics that specifically target or block these interactions.

### FMT

Fecal microbiota transplantation (FMT) has long been recognized for its remarkable efficacy in restoring microbial balance in patients suffering from *Clostridioides difficile* infection [30]. Given the extensive dysbiosis observed in many cancer contexts, it is plausible that FMT could similarly reestablish microbial homeostasis and, in turn, help mitigate disease progression.

The recognition of microbial interactions as central players in cancer biology emerged from pioneering studies by Iida et al. and Sivan et al., who first demonstrated that perturbations to the gut microbiome can alter tumor progression and responsiveness to therapeutic interventions [31, 32]. These foundational findings prompted a new line of investigation into whether FMT itself could modulate cancer outcomes, and in 2015 Vetizou et al. published the first pre-clinical work examining anti-CTLA-4 effectiveness supplemented with FMT [33].

In 2018, three seminal studies by Gopalakrishnan et al.., Matson et al., and Routy et al. sparked global interest in FMT as a potential adjunct to anti-PD-1 cancer immunotherapy. Gopalakrishnan et al. profiled both the oral and gut microbiomes of melanoma patients undergoing anti-PD-1 therapy and identified striking compositional differences between responders and non-responders [34]. Distinct bacterial pathway signatures were associated with effective anti-tumor immunity. When stool from responder patients was transplanted into germ-free mice prior to melanoma induction, only those receiving responder-derived material exhibited an enhanced anti-tumor effect. These mice demonstrated increased CD8 + T-cell infiltration in the tumor microenvironment and overall elevated CD45 + cell abundance in the gut, suggesting that species within the families *Ruminococcaceae* and *Faecalibacterium* promote anti-tumor immunity through immunomodulatory mechanisms.

In parallel, Routy et al. performed a similar investigation based on the observation that resistance to immune checkpoint inhibitors correlates with gut microbiome perturbations [35]. In antibiotic-treated or germ-free mouse models, fecal material from immune checkpoint inhibitor (ICI)-responsive patients reinstated sensitivity of PD-1 blockade, whereas transplants from non-responders did not. This study identified *Akkermansia muciniphila* as a key microbial mediator capable of restoring ICI efficacy, even when administered as a single bacterial supplement. Mechanistically, enhanced recruitment of CD4 + T-cells expressing CXCR3 and CCR9 was observed, driven by increased interleuken-12 secretion from local dendritic cells.

In a very similar study, Matson et al., identified further species abundant in ICI responders including *Bifodobacterium longum*,* Collinsella aerofaciens*, and *Enterococcus faecium* [36]. Although the immune subsets and bacterial taxa differed between the two studies, their overarching conclusion was consistent: the composition of the gut microbiome profoundly influences immunotherapy responsiveness, and microbiome modulation via FMT can restore anti-tumor efficacy through immune reprogramming.

**Fig. 2:**
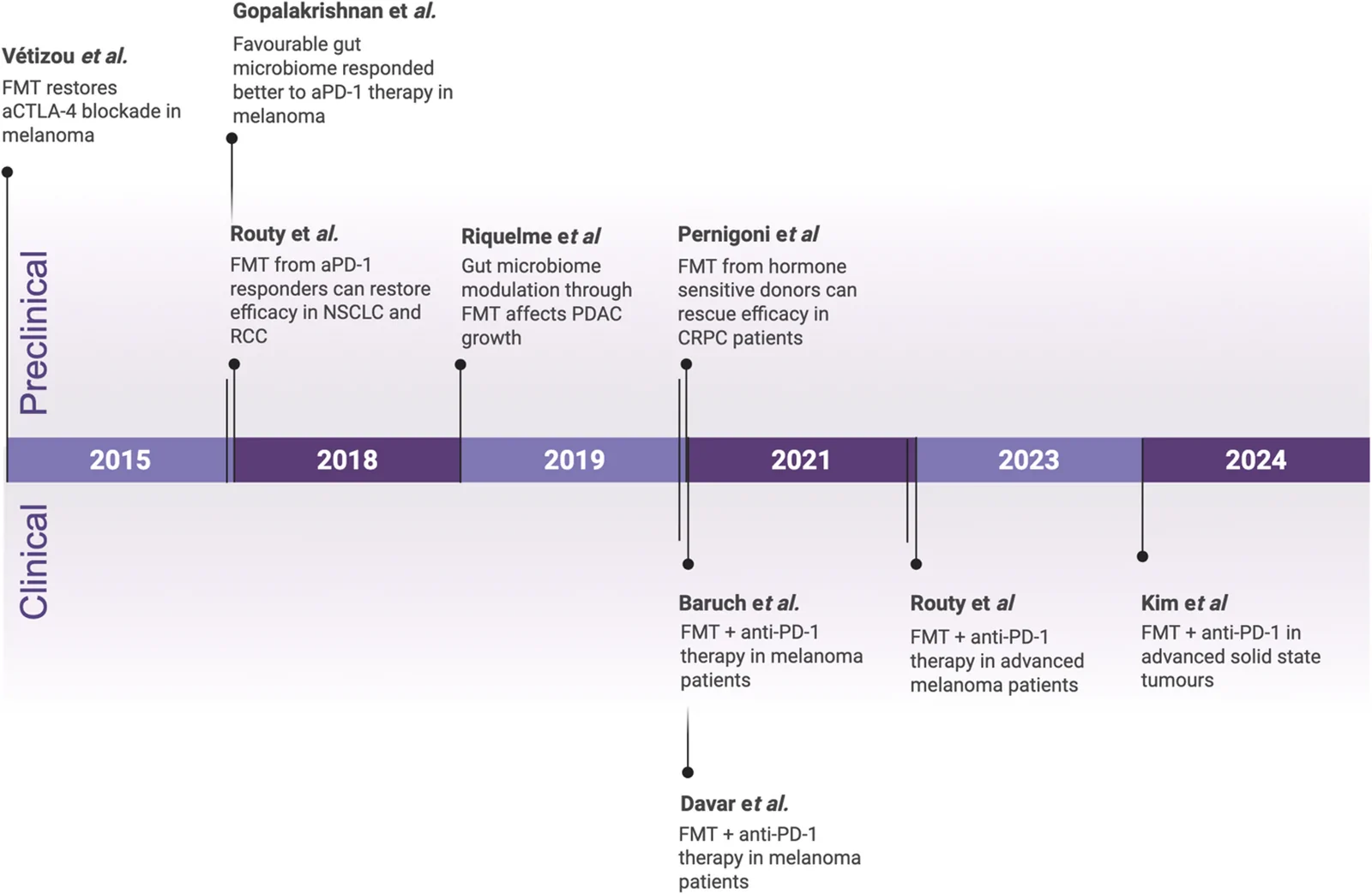
Time-line of recent FMT clinical and preclinical discoveries: highlighted significant advancements in understanding of FMT in both preclinical in vivo studies and more recent human clinical trials

Following these landmark discoveries (Fig. 2.), multiple studies extended FMT investigation beyond melanoma to other malignancies. In pancreatic adenocarcinoma, specific bacterial signatures distinguished patients with long-term survival, and transplantation of stool from these patients into mice conferred improved disease outcomes [37]. In castration-resistant prostate cancer, gut microbes were shown to produce androgenic metabolites that sustain tumor growth; correspondingly, FMT from hormone-sensitive donors attenuated tumor progression [38]. Lastly, FMT from hepatocellular carcinoma patients accelerated disease in murine models, exacerbating inflammation and fibrosis and revealing bacterial translocation from the gut to the liver [39]. Although these studies differ in cancer type and outcome, collectively they underscore the utility of FMT as a tool to interrogate the microbiome’s causal role in oncogenesis and therapy resistance.

Building on these preclinical successes, two independent phase I clinical trials, conducted by Baruch et al. and Davar et al., introduced FMT into the human oncology setting [40, 41]. In the study performed by Baruch et al., ten metastatic melanoma patients who were refractory to anti-PD-1 therapy received FMT from prior responders. Three of the ten achieved objective clinical responses (one complete and two partial according to iRECIST criteria). Although microbial analyses did not pinpoint a single determinant species, multiple taxa previously associated with favorable immunotherapy outcomes were enriched in responders. Furthermore, transcriptomic profiling revealed upregulation of antigen-presentation pathways and other immune-related genes in tumors from FMT responders.

Similarly, Davar et al. performed FMT in fifteen patients with advanced melanoma who were refractory to ICIs, observing clinical benefit in six individuals. Post-FMT, alpha diversity increased across all participants, with responders demonstrating higher engraftment of donor-derived species. Responders also exhibited elevated circulating CD8 + T cells with activation maker expression, alongside distinct increased pro-inflammatory cytokines in the peripheral blood. Additionally, distinct serum metabolomic profiles enriched in secondary bile acids, triacylglycerols, and other metabolites previously linked to ICI responsiveness provided complementary insight into treatment response mechanisms.

Together, these early clinical trials established a foundation for continued exploration of FMT as a therapeutic strategy in oncology. Despite converging evidence implicating cytotoxic T cells and immune reactivation in mediating FMT benefit, the precise molecular and cellular mechanisms linking microbial cues to tumor immune modulation remain incompletely understood. Moreover, the heterogeneity in donor composition, patient responses, and study designs highlights the immense complexity of this therapeutic approach, suggesting that FMT efficacy likely arises from a multifaceted network of microbial and host interactions that are only beginning to be unraveled.

Interestingly, since the publication of these cancer-focused FMT clinical trials in 2021, very few additional reports, aside from safety-focused studies, have presented clinical outcome data [42–44], until the end of 2025. In contrast, more than thirty registered clinical trials are currently investigating the efficacy of FMT in cancer [45]. This discrepancy between the number of ongoing studies and the limited availability of published results likely reflects both the scientific and logistical challenges inherent to this field, as well as our still-evolving understanding of the microbiome’s complex and context-dependent role in cancer biology.

In January 2026, three clinical trials were reported evaluating the combination of immune checkpoint inhibition with FMT across an expanded spectrum of malignancies [46–48]. Two studies investigated metastatic renal cell carcinoma (mRCC), while the third extended combination strategies in melanoma and incorporated non-small cell lung cancer (NSCLC). Objective responses were observed in subsets of patients across all trials, indicating that microbiome modulation may have broader relevance beyond melanoma. However, response heterogeneity remained substantial, underscoring the incomplete understanding of the determinants of therapeutic benefit.

Importantly, two of the three trials diverged from earlier melanoma studies in that FMT material was derived from healthy donors rather than from ICI-responsive cancer patients. This distinction challenges the prevailing assumption that therapeutic efficacy requires transfer of a responder-specific, immunologically primed microbial consortium. If healthy donor stool can potentiate ICI response, then the mechanism of benefit may not depend solely on the presence of discrete pro-immunogenetic taxa enriched in these donors. Rather, these findings raise the possibility that FMT exerts benefit through broader ecological remodeling, immune recalibration, or removal of inhibitory microbial configurations. Consistent with this reinterpretation, the FMT-LUMINate trial reported no obvious requirement for sustained donor-recipient microbiome convergence at endpoint. Instead, clinical benefit correlated with depletion of bacterial strains associated with poor outcomes. These findings challenge the prevailing donor-centric paradigm that has guided much of the field, and suggests that therapeutic success may depend less on engraftment of specific beneficial organisms and more on a disruption of pathogenetic microbial networks within the recipient. Collectively, this data suggests a potential conceptual shift: from a donor-centric model focused on taxonomic transfer, to a recipient-centric model emphasizing ecological susceptibility.

FMT remains a challenging therapeutic strategy to advance clinically. Standardization between samples is nearly impossible, resulting in each recipient receiving a compositionally distinct microbial community. Donor variability further complicates outcomes, as differences in baseline microbiota influence engraftment efficiency and raise questions about donor-recipient compatibility [49]. In addition, antibiotic use, which is often necessary during cancer treatment, can substantially alter the success of FMT and introduce unpredictable variation into the therapeutic responses [50]. Furthermore, one study even showed that the specific antibiotic, vancomycin, when used in pretreatment lead to worse patient outcomes [51]. The persistent inability to explain why some patients respond to FMT while others do not, even when receiving material from the same donor, underscores the need for robust predictive biomarkers to identify optimal donors and ideal candidates for transplantation. With improved stratification tools, future clinical studies may be able to evaluate FMT across a broader range of cancer types. At the same time, these limitations highlight the appeal of more controlled, reductionist approaches, paving the way for strategies centered on defined bacterial supplementation or single-strain therapeutics.

### Bacterial supplementation and monoculture-based strategies

Although FMT has produced promising early results as a combinatorial cancer therapy, its practical limitations, including variability in donor material, challenges in standardization, and regulatory hurdles, may restrict its broader clinical adoption. If the therapeutic benefits of FMT can be attributed to specific bacterial taxa, then targeted delivery of single, well-defined strains could represent a next-generation more controllable approach. The use of probiotics, such as *Lactobacillus and Bifidobacterium* to support gut and immune health is well established; however, their current application is affixed to general preventative health, and their use as targeted cancer therapeutics has only recently begun to be explored [52].

Many monoculture-based studies were motivated by findings from FMT research. In the 2018 melanoma study by Routy et al., the authors demonstrated that resistance to anti-PD-1 therapy was influenced by the gut microbiome and identified *Akkermansia muciniphila* as a key species associated with restored ICI responsiveness [35]. Follow-up experiments confirmed that oral administration of *A. muciniphila* recapitulated immunomodulatory features of FMT and enhanced therapeutic efficacy when combined with anti-PD-1 therapy, suggesting that single-strain supplementation can reproduce complex FMT-derived benefits. However, broader evidence across the field indicates that therapeutic effects may be dose-dependent; microbial abundance and ecological context likely influence functional outcomes. Indeed, in a subsequent study from the same group, a relative *Akkermansia* abundance of approximately 4.8% in stool samples was associated with ICI resistance, underscoring that deviations from an optimal range may attenuate or even reverse beneficial effects [27]. Taken together, these findings underscore that while single-species supplementation can mimic certain FMT-derived immunomodulatory effects, therapeutic efficacy is likely highly context- and dose-dependent, highlighting the need for precise quantification and ecological consideration of microbial interventions rather than assuming uniformly beneficial effects of individual taxa.

Additional work has shown similar outcomes with *Bifidobacterium*. Supplementation with two strains of *Bifidobacterium breve* reduced MC38 colorectal tumor burden in mouse models [53]. Immune profiling revealed increased intratumoral CD4 + and CD8 + T-cell infiltration, decreased Tregs, and elevated IL-2 and IFN-y, with further tumor reduction when combined with anti-PD-1. Furthermore, a clinical trial using a bifidogenic live bacteria in combination with anti-PD-1 immunotherapy, which observed higher objective response rates in patients receiving this single strain therapeutic [54, 55].

While host immune responses to bacterial therapies are often well characterized, the bacterial mechanisms underlying these effects remain less clear. Microbial ecology is complex, and tools for dissecting bacterial signaling pathways lag far behind those available for mammalian systems. This gap makes it challenging to map how individual bacterial species initiate immune-modulatory or anti-tumor effects.

An illustrative example in which both components are clearly elucidated involves the use of *Clostridium butyricum* as a probiotic to enhance ICI sensitivity in colorectal cancer, a tumor type typically resistant to ICIs [56]. Supplementation increased CD8 + T-cell activity and reduced TAM-mediated immunosuppression. Notably, the authors identified a mechanistic link: the bacterial surface protein SecD interacted with the host receptor GPR78, disrupting PI3K-AKT-NF-kB signalling, reducing IL-6 secretion, and limiting TAM recruitment. Such studies highlight the need and potential for deeper mechanistic insight into microbe-host interactions in cancer therapy. Further work with *C. butyricum*, specifically the strain CBM588, is beginning to be explored in clinical trials alongside treatment with ICI therapies [55], however results so far are quite preliminary and require further investigation to conclude efficacy.

Defined bacterial consortia offer a middle ground between single-strain supplementation and whole-community FMT. In one study, an orally delivered four-strain *Clostridiales* consortium reduced colorectal tumor growth independently of ICI therapy and provided prophylactic benefit [57]. Tumor suppression was attributed to enhanced infiltration of IFN-y + and granzyme B+ CD8 + T cells, although the specific bacterial contribution remained unclear. However, in a follow-up study, metabolomic analysis revealed elevated levels of the medium-chain fatty acid 3-hydroxy-dodecanoic acid, which alone was sufficient to suppress tumor growth in mouse models [58]. Supporting the idea of multi-strain administration as a therapeutic, one clinical trial administered a consortia of bacteria combined with anti-PD-1 therapy to test the clinical efficacy of such therapeutics and found that primary safety outcomes were achieved [59].

These findings underscore the possibility that bacterial metabolites, rather than the microbes themselves or their membrane bound surface proteins, may constitute the active therapeutic agents. From a medicinal perspective, identifying a small molecule and its corresponding target is far more attractive as a therapeutic strategy than the much more complex approach of administering unstandardized FMT material or live bacterial strains.

Finally, rather than applying bacteria or bacterial consortia directly, their bioactive metabolites – once identified – can be supplemented themselves as a therapeutic. Often arising from studies of individual bacteria species, the discovery of such active metabolites can enable either the direct use of the molecule itself, or the development of derivative small-molecule drugs based on its chemical scaffold. This approach has been shown in multiple studies targeting various cancer types [60, 61].

This concept is reinforced by studies demonstrating that microbial metabolites can also drive tumor-promoting, rather than tumor-suppressive phenotypes. For example, indole-producing gut bacteria were shown to accelerate pancreatic cancer growth via TAM-mediated immunosuppression through aryl-hydrocarbon receptor signalling [22]. Even when microbial effects are detrimental [62], identifying specific metabolite-receptor axes reveals actionable therapeutic targets and underscores that bacterial influence can extend beyond the gastrointestinal tract.

Moreover from the perspective of tryptophan-derived based bacterial metabolites, the molecule indole-3-acetic acid (3-IAA) was found to be enriched in pancreatic ductal adenocarcinoma patients who responded to chemotherapy [63], illustrating how similar research topics can yield markedly different conclusions. 3-IAA was found to increase reactive oxygen species levels via neutrophil myeloperoxidase activity, thereby reducing overall metabolic function and proliferative capacity of the proximal cancer cells. Together, these two studies highlight the complexity and diversity of microbiome-mediated effects; however, it must be mentioned that in the previous study effects were seen under dietary tryptophan-free experimental conditions, whereas in patients the increased chemotherapy efficacy was observed during steady-state conditions, highlighting the importance of context-dependent conclusive results.

**Fig. 3:**
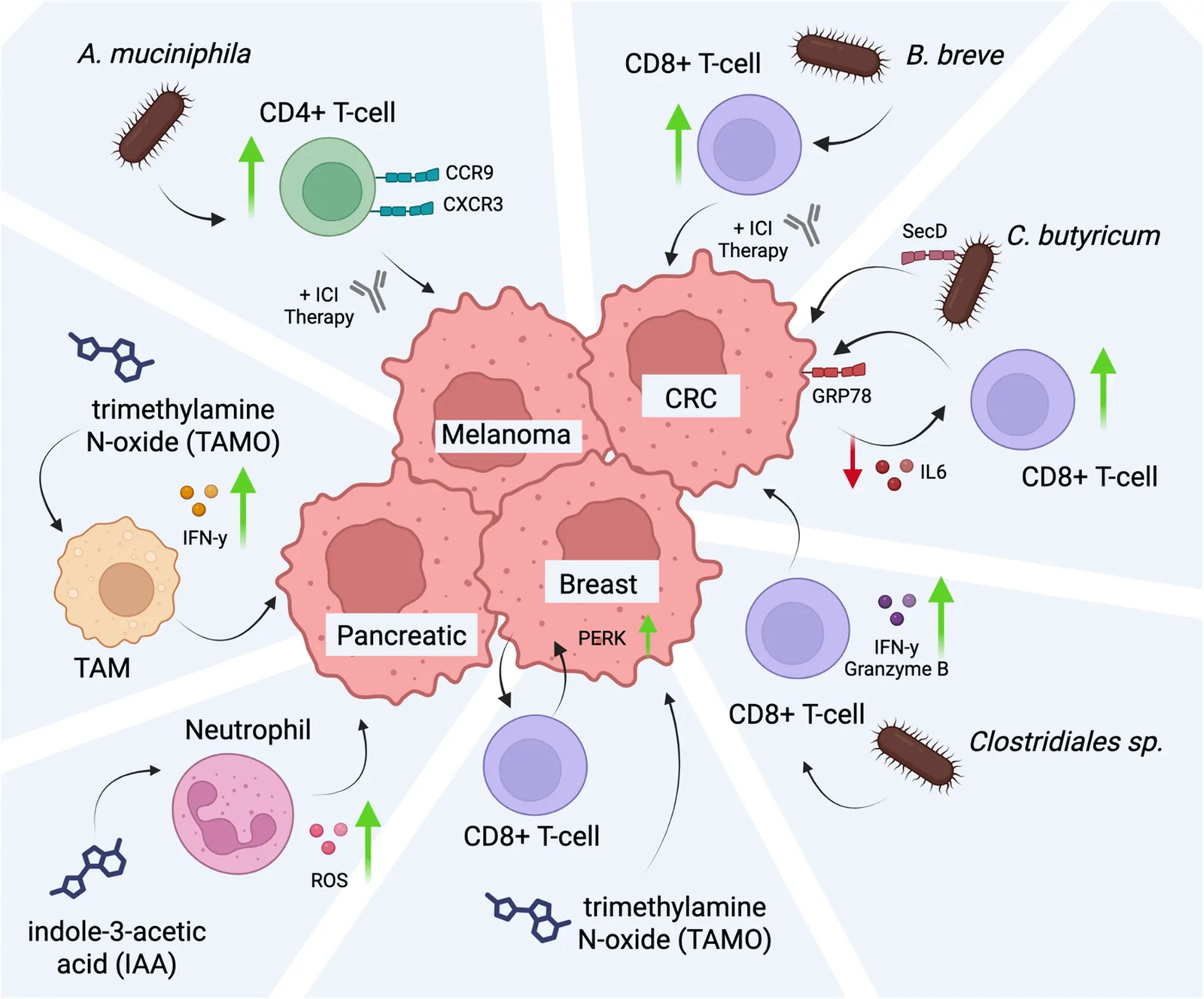
Bacterial influence on anti-cancer immunity: elucidated examples ([35, 53, 56, 57, 60, 61, 63]) of the influence of bacteria or their secreted counterparts in inducing anti-cancer efficacy

A persistent challenge in the field is the inconsistency observed across studies investigating similar bacterial species. For instance, while early work by Vetizou et al. reported *Bacteroides fragilis* as enhancing anti-CTLA-4 responses [33], several other studies have since implicated *B. fragilis* in tumor promotion across multiple cancer types [64, 65]. Such contradictions raise important questions: Can a single bacterial species truly exert broad, systemic anti-tumor effects? Or are these observations highly context-dependent, shaped by strain-level variation, host genetics, and other yet unidentified factors? Increasingly, it appears that no single determinant fully accounts for these phenomena; rather, we are gradually piecing together a large complex narrative of how the microbiome affects cancer progression (Fig. 3.). As mechanistic pathways are uncovered for individual strains, a more coherent picture will likely emerge, one in which multiple microbial and host factors converge to influence therapeutic outcomes.

### Engineered bacteria

While single-strain supplementation and defined bacterial consortia provide valuable insights into how individual taxa or metabolites can influence tumor immunity, these approaches are ultimately constrained by our limited understanding of the entire host-microbe interactions. Another emerging direction in bacteria-based cancer therapeutics is the use of genetically modified strains engineered for targeted tumor killing. Enabling the controlled delivery of targeted molecules, engineered bacteria may allow for a modifiable system for next generation therapeutics.

One of the most extensively explored organisms in this field is the nonpathogenic *Escherichia coli* Nissle 1917 (EcN), which can be genetically modified to deliver therapeutic payloads directly within the tumor microenvironment. In several studies, EcN was engineered to secrete immune-stimulatory cytokines, including IL-2 and IFN-y, enabling localized cytokine release after intratumoral injection [66, 67]. This strategy avoids systemic toxicities associated with recombinant cytokine therapy while supporting endogenous anti-tumor immunity through enhanced recruitment and activation of cytotoxic immune cells. Additionally, bacterial in situ cytokine production reduces the need for repeated dosing and drastically lowers the cost associated with purified protein therapeutics.

More sophisticated engineering advances have further expanded this concept. A recent study designed EcN to produce and deliver neoepitope-containing peptides to the tumor, resulting in increased antigen-presenting cell recruitment and elevated intratumoral CD4 + and CD8 + T-cell responses [68]. These findings indicate that engineered bacteria can function as in situ vaccine platforms capable of reshaping the tumor immune landscape.

Other vectors than *E.coli Nissle 1917* have been explored as well in this context, such as attenuated *Salmonella typhimurium* [69]. The bacteria, expressing neoantigens specific to anti-tumor immunity, used intrinsic tumor infiltrating characteristics to promote a higher immune infiltration into the TME.

Engineered bacterial therapies have progressed beyond preclinical development and into human clinical testing. The anaerobic, non-toxic *Clostridium novyi-*NT strain underwent first in-human testing in 2021 for advanced solid tumors, with approximately 80% of evaluable patients achieving stable disease [70]. A follow-up study in 2025 combined *C. novyi-*NT with the immunotherapy pembrolizumab, demonstrating a manageable safety profile and achieving three partial responses and one complete response [71]. These findings represent some of the earliest clinical evidence supporting engineered bacterial cancer therapy in humans.

Innovative delivery technologies have also emerged. One strategy involves administering bacteria containing iron nanocomposites that accumulate in solid tumors [72]. Upon exposure to an external magnetic field, these particles generate localized heat, lysing the bacterial carrier and releasing anti-CD47 nanobodies. The combination of bacterial debris and targeted CD47 blockade leads to robust immune activation and recruitment of effector immune cells into the tumor microenvironment.

Together, these studies highlight the potential of engineered microbes as a new class of cancer therapeutics, integrating synthetic biology with immuno-oncology and offering programmable, tumor-targeted, and cost-effective treatment modalities. Their capacity to remodel the tumor immune landscape underscores their value as both therapeutic agents and biological delivery systems.

## Integration with conventional cancer therapies

One major area of investigation in microbiome-cancer research concerns how microbial communities influence the efficacy of established cancer therapies. Although evidence that microbial modulation alone can reliably reduce tumor burden is still emerging, recent studies increasingly suggest that the microbiome can enhance, impair, or otherwise reshape responses to a range of therapeutic modalities. Accordingly, many current efforts focus on how altering the microbiome interacts with, and potentially augments existing treatments. While several of the aforementioned studies have already explored microbiome-ICI reactions, and there are many others that examine this phenomenon [73–75], this section highlights additional work examining how microbial modulation interfaces with other cancer therapies.

A preclinical study in epithelial ovarian cancer investigated the impact of antibiotic-induced gut dysbiosis on tumor progression during treatment with the platinum-based chemotherapy agent cisplatin [76]. Disruption of the gut microbiota accelerated tumor growth and reduced sensitivity to cisplatin. Mechanistically, tumors from antibiotic-treated animals exhibited decreased apoptosis, reduced DNA damage, and increased angiogenesis, suggesting that microbial loss may alter host pathways involved in therapeutic response. Notably, the study did not identify specific bacterial taxa or metabolites mediating these effects, leaving open the question of whether the phenotype results from depletion of particular organisms or from a generalized reduction in microbial biomass leading to systemic immunometabolism dysfunction.

It is worth emphasizing that, like most microbiome studies, this work focused on the gut microbiota’s influence on a distal organ system. The scarcity of studies investigating the vaginal microbiome and its role in gynecologic malignancies remains particularly striking given the substantial microbial niche and clinical burden of these cancers. However, it is of particular interest that the gut microbiome is able to influence its effects to such distal organs and remains an area of great interest.

Another compelling example arises from prostate cancer, where the gut microbiome appears to influence recurrence of castration-resistance disease [38]. Despite elimination of endogenous androgens using standard hormone therapy interventions, patients who developed castration-resistant prostate cancer harbored gut microbial communities capable of producing androgenic metabolites. FMT from hormone-sensitive donors restored treatment responsiveness in initially refractory recipients. Furthermore, *Prevotella stercorea* was found to be enriched in hormone-sensitive patients, and supplementation with this organism similarly restored sensitivity to androgen deprivation therapy. Beyond its therapeutic implications, this study highlights the potential for microbial taxa or their metabolites to serve as biomarkers guiding clinical decision-making. It also suggests that microbial modulation of hormone pathways, a central feature of many cancer types, may represent a broader, underexplored mechanisms linking the microbiome to tumor progression.

In addition to chemotherapy and hormonal therapies, microbial products have also been shown to modulate responses to cellular immunotherapies. Although not an immune-checkpoint inhibitor, chimeric antigen receptor (CAR) T cell therapy has demonstrated susceptibility to microbiome-derived signals. In one study, CD8 + T cells and CAR T cells were pulsed with the short-chain fatty acids (SCFAs) pentanoate and butyrate, metabolites produced by gut commensals such as *Faecalibacterium prausnitzii* and *Anaerostipes hadrus* [77]. These SCFAs enhanced effector function through histone deacetylase inhibition, epigenetic remodeling, and activation of mTOR signalling, ultimately increasing IFN-y and TNF-a production. In vivo, SCFA-treated effector cells demonstrated improved anti-tumor activity, illustrating how microbial metabolites can be leveraged to potentiate cellular therapies. This example underscores a growing recognition that microbial products, independent of live bacteria, can serve as powerful modulators of anti-tumor immunity and result from knowledge gained from the study of the microbiome.

From a deleterious perspective, and interesting study examined the features of the gut microbiome and its ability to affect chemoradiation response in patients with cervical cancer [78]. *Lactobacillus iners* was able to induce treatment resistance through the production of L-lactate, which led to downstream metabolic changes in tumors and a subsequent increase in tumor aggressiveness. Awareness and utilization of this species as a biomarker could therefore help guide treatment strategies, as inhibitors targeting lactate pathways already exist and are utilized in cancer therapy.

Collectively, these studies emphasize that the microbiome serves as an important determinant of therapeutic efficacy across diverse treatment classes—including chemotherapy, hormonal therapy, and cellular immunotherapy. Rather than acting as isolated modulators, microbial taxa and their metabolites appear to intersect with core oncologic pathways such as DNA damage responses, hormone biosynthesis, angiogenesis, and immune effector programming. As a result, the integration of microbial modulation with conventional cancer treatments offers a promising avenue to improve response durability, overcome resistance, and personalize therapeutic strategies. Continued mechanistic work will be crucial for distinguishing causal microbial drivers from correlative signals, but the current evidence strongly suggests that targeting the microbiome may become an increasingly important component of combination cancer therapy design.

## Challenges and limitations

The field of microbiology in the context of human cancers remains a relatively young discipline, and our understanding of these complex interactions continues to evolve. Substantial knowledge gaps persist, not only regarding the underlying biological mechanisms involved but also in the methodological tools available to investigate them. As mentioned above, reliable high-throughput methods for detecting live bacteria in human samples are still lacking; most current techniques bind to specific antigens that could be fragmented at the time of detection and provide limited information about viability, function, and ecological relevance in the tumor microenvironment [79]. Additionally, the temporal dynamics of bacteria translocation, specifically when and how microbiomes reach tumor sites, remain largely unknown. Some current hypotheses as to how this is being achieved accredit phagocytizing immune cells, and possible circulating tumor cells [80].

Although this review has focused primarily on bacteria, it is important to acknowledge that the human microbiome is composed of diverse microbial kingdoms. Fungi, viruses, archaea, and other microorganisms also inhabit our mucosal and tumor niches and may contribute meaningfully to cancer progression or therapy response [81]. Integrating these additional microbial players introduces further complexity but is essential for developing a more complete picture of host-microbe interactions in oncology. At the same time, novel mechanisms of bacterial influence are beginning to emerge beyond immune reprogramming and metabolic rewiring, including epigenetic modification of host cells, which adds yet another layer of intricacy to the field [82].

Finally, while many studies to date have centered on how microbial modulation influences cancer progression directly, a paralleled body of research examines the microbiome’s role in shaping secondary complications of cancer and its treatments. Microbial interventions have been investigated for their potential to mitigate common therapy-associated toxicities, improve patient quality of life, and enhance overall treatment tolerability [83]. These avenues illustrate how broadly the microbiome interfaces with cancer care, and how many unanswered questions still limit translation into the clinic.

In summary, although enthusiasm for microbiome-based oncology is rapidly growing, significant scientific and technical barriers must still be addressed. Continued development of precise detection tools, integration of multi-kingdom microbiome data, and deeper mechanistic studies will be essential for transforming early observations into reliable, standardized, and clinically actionable therapies.

## Conclusion and future directions

The integration of the microbiome into the study of cancer biology represents a rapidly emerging frontier, offering new perspectives on tumorigenesis, therapeutic response, and personalized medicine. Although still in its early stages, research in this area has already revealed that both intratumoral and gut-resident bacteria can shape cancer progression through diverse mechanisms, ranging from immune modulation to metabolic reprogramming. Tumors across multiple tissue types harbor distinct microbial ecosystems, and growing evidence suggests that these communities play a functional role in cancer development and treatment response.

As studies continue to uncover mechanistic links between microbes and key oncogenic pathways, the potential for harnessing bacteria and their products as therapeutic tools becomes increasingly tangible. Microbial signatures also hold promise as biomarkers for predicting disease risk, stratifying patients, and guiding treatment decisions. Yet, translating these insights into clinical implementation requires a more complete understanding of which bacterial taxa are causally involved, how they exert their effects, and how they vary across individuals, tumor types, and treatments.

Looking ahead, the challenge lies not only in discovering additional microbial contributors to cancer but also in determining how to manipulate these organisms safely and effectively for therapeutic gain. This emerging mechanistic clarity naturally motivates the next area of investigation: whether individual bacterial species, when administered intentionally, can recapitulate the beneficial effects observed in broader microbial modulation approaches.

## Data Availability

No new data generated as part of this review article.
